# Risk factors for poorer health literacy in patients with liver cirrhosis

**DOI:** 10.1371/journal.pone.0255349

**Published:** 2021-07-27

**Authors:** Leonard Kaps, Katharina Hildebrand, Michael Nagel, Maurice Michel, Wolfgang Maximilian Kremer, Max Hilscher, Peter R. Galle, Jörn M. Schattenberg, Marcus-Alexander Wörns, Christian Labenz

**Affiliations:** 1 Department of Internal Medicine I, University Medical Centre of the Johannes Gutenberg-University, Mainz, Germany; 2 Cirrhosis Centre Mainz (CCM), University Medical Centre of the Johannes Gutenberg-University, Mainz, Germany; 3 Institute of Translational Immunology, University Medical Centre of the Johannes Gutenberg-University, Mainz, Germany; Center for Primary Care and Public Health, SWITZERLAND

## Abstract

**Background:**

Health literacy is a concept that refers to patients’ ability to manage their disease and the health system’s ability to guarantee access to services. There is evidence that health literacy impacts the health outcomes of patients with chronic diseases, but detailed information on this topic in patients with liver cirrhosis is scarce. It was the aim of this study to identify risk factors for poorer health literacy in patients with liver cirrhosis.

**Methods:**

89 patients with liver cirrhosis were enrolled in this study and health literacy was measured using the Health Literacy Questionnaire (HLQ). Covert hepatic encephalopathy (CHE) was diagnosed clinically according to the West-Haven Criteria (HE grade 1) and the PHES (minimal HE). Depressive symptoms were assessed using the Hamilton Depression Rating Scale (HDRS). Based on the nine subscales of the HLQ, risk factors for poor health literacy were identified using linear regression models.

**Results:**

Normalized HLQ scores ranged between 65–76%, while appraisal of health information had lowest score (65%) and ability to actively engage with healthcare providers had highest score (76%). Multivariable regression analyses revealed an association of poorer health literacy and liver function as determined by MELD score and complications of liver cirrhosis such as a history of ascites or CHE. Additionally, we identified modifiable or preventable factors such as depressive symptoms, a history of falls, and active smoking as risk factors for poorer health literacy.

**Conclusion:**

Multiple factors seem to impact on health literacy in patients with liver cirrhosis. Addressing modifiable and preventable factors may improve health literacy.

## Introduction

Modern healthcare systems tend to be more and more complex; consequently it may be challenging for patients to manage their diseases and receive optimal healthcare [1, 2]. Health literacy is a concept that covers key determinants of patient’s ability to manage their disease and the health system’s ability to guarantee access to services [3]. The world health organization (WHO) summarizes health literacy as “the cognitive and social skills which determine the motivation and ability of individuals to gain access to, understand and use information in ways which promote and maintain good health” [4]. There is evidence that low health literacy is associated with increased hospitalization, health system costs, and mortality, with insufficient adherence to healthcare services, medical check-ups, and prescribed medications, and finally with difficulties to communicate with healthcare professionals and poor knowledge about disease processes [5–12]. Insufficient health literacy may especially impact on patients with chronic diseases such as liver cirrhosis, which demand ongoing support by their healthcare providers [1, 12, 13]. Liver cirrhosis is the end-stage of almost all chronic liver diseases and is a major cause of morbidity and mortality. Additionally, liver cirrhosis represents a significant health problem, causing substantial economic burden for the healthcare system and society [14]. Beside life-threating complications like variceal bleeding due to portal hypertension or liver cancer (hepatocellular carcinoma), patients with liver cirrhosis may suffer from hepatic encephalopathy (HE), which even in its lowest grade may impair patients’ quality of life [15].

Previous studies have demonstrated that patients with liver cirrhosis show insufficient understanding and knowledge about their disease suggesting that these patients may be at risk for poor health literacy [8, 16, 17]. Recently, Grydgaard et al. found poor health literacy in outpatients with liver cirrhosis in a tertiary care hospital in Denmark and concluded that these patients need intensified support in their disease management [16]. However, only parts of a validated health literacy questionnaire have been used in this study (exclusively conducted in Denmark) and potentially modifiable and preventable risk factors for poorer health literacy have not been studied extensively. Therefore, aims of this study were 1) to investigate health literacy in German patients with liver cirrhosis and 2) to identify potential risk factors for poorer health literacy.

## Patients and methods

### Patients

89 in- and outpatients with liver cirrhosis were included into this prospective, exploratory study between January 2019 and November 2020 at the Cirrhosis Centre Mainz (CCM) that takes part of the Department of Internal Medicine I of the University Medical Centre of the Johannes Gutenberg-University in Mainz, Germany. The leading aetiology of underlying liver disease was determined according to clinical, serological and histological findings. Diagnosis of liver cirrhosis was made by histology, typical appearance in ultrasound or radiological imaging, endoscopic features of portal hypertension, and medical history. Detailed medical history was taken for each patient including social anamnesis, disease history and history of falls during the previous six months. Blood biochemistry was performed in all patients. Model of end-stage liver disease (MELD) and Child-Pugh (CP) score were calculated to determine the severity of liver disease. Patients were not approached for the study if they fulfilled one or more of the following criteria: chronic alcohol consumption at study inclusion, the presence of pre-terminal comorbidities (e.g. New York Heart Association (NYHA) III-IV, chronic obstructive pulmonary disease (Global Initiative for Chronic Obstructive Lung Disease–Gold) C and D), presence of hepatocellular carcinoma (HCC) or other active malignancies, active infection or severe neurological comorbidities (i.e. dementia or history of stroke).

### Diagnosis of HE

Diagnosis of covert HE (CHE) was established as recently described [18]. At first, every patient was examined by an experienced hepatologist to rule out OHE. As recommended by current guidelines, hepatic encephalopathy grade 1 (HE1) was diagnosed after detailed neurological examination according to the West-Haven criteria based on findings like euphoria, anxiety, lack of awareness, impaired performance of addition and/or shortened attention span [19]. Afterwards, portosystemic encephalopathy (PSE) syndrome test that produces the PHES (consisting of the number connection test-A [NCT-A], the number connection test-B [NCT-B], the digit symbol test [DST], the serial dotting test [SDT], and the line tracing test [LTT]) was performed in all patients (apart from 7 patients who refused to conduct the PHES). Interpretation of PHES was done as previously described with German norms [20]. All tests were conducted by trained medical staff and performed in a quiet, lighted room between 09.00 a.m. and 04.00 p.m.. A score <-4 was considered as pathologically.

### Assessment of health literacy

Health literacy was assessed using the Health Literacy Questionnaire (HLQ), which was validated in the German version in 2017 [5, 21]. The HLQ contains 44 items, which are divided into nine areas of health literacy [5]. The first five scales are scored on a 4-point Likert scale (ranging from strongly disagree to disagree, agree, and strongly agree), building part I. The other four scales, representing part II, are scored on a 5-point Likert scale where respondents are asked to rate the level of difficulty in undertaking a task (ranging from cannot do, always difficult, usually difficult, sometime difficult, usually easy, and always easy). Higher scores indicate better health literacy.

The scales are subdivided into the following categories:

1. Feeling understood and supported by healthcare providers (HPS) (4 items),
2. Having sufficient information to manage my health (HSI) (4 items),
3. Actively managing my health (AMH) (5 items),
4. Social support for health (SS) (5 items),
5. Appraisal of health information (CA) (5 items),
6. Ability to actively engage with healthcare providers (AE) (5 items),
7. Navigating the healthcare system (NHS) (6 items),
8. Ability to find good health information (FHI) (5 items),
9. Understanding health information well enough to know what to do (UHI) (5 items).

Licence of the questionnaire was granted by the Swinburne University, Hawthorn, Australia. A trained healthcare professional assisted the patients to complete a paper version of the questionnaire.

### Assessment of depressive symptoms

Depressive symptoms were assessed using the Hamilton Depression Rating Scale (HDRS) [22]. The HDRS is a multi-item questionnaire and was designed for adults. It includes items addressing mood, feelings of guilt, suicide ideation, insomnia, agitation or retardation, anxiety, weight loss, and somatic symptoms. In total, the HDRS contains 17 items scored either on a 3-point or 5-point Likert scale. A score of 0–8 is considered to be normal, while 9–16 points indicate minor, 17–24 points moderate, and ≥25 points severe depressive symptoms [22].

### Ethics

The study was conducted according to the ethical guidelines of the 1975 Declaration of Helsinki and its later amendments. The study protocol was approved by the ethics committee of the Landesärztekammer Rhineland-Palatine (Nr. 2019–14483). Written informed consent was obtained from every participant.

### Statistical analysis

Quantitative data are expressed as medians with interquartile ranges (IQR). Categorical variables are given as frequencies and percentages, respectively. First, the correlation of demographic and clinical variables with the raw scores of the sub-scales of the HLQ were assessed by means of univariable analyses. Correlation analyses were conducted using Spearman’s rank correlation and point-biserial correlation. Variables with p <0.1 in the univariable analysis were subsequently considered in a multivariable linear regression model for each sub-scale. To reliably identify factors being associated with poorer health literacy, the final multivariable models were built based on a stepwise variable selection procedure for each scale. Only patients with complete datasets were included into the respective analyses.

Our complete data analysis is exploratory. Hence, no adjustments for multiple testing were performed. For all tests we used a 0.05 level to define statistically relevant deviations from the respective null hypothesis. However, due to the large number of tests, p-values should be interpreted with caution. Data were analysed using IBM SPSS Statistic Version 25.0 (Armonk, NY: IBM Corp.). Figure was drawn with GraphPad Prism Version 8.0.2 (GraphPad Software, California, US).

## Results

**Fig 1:**
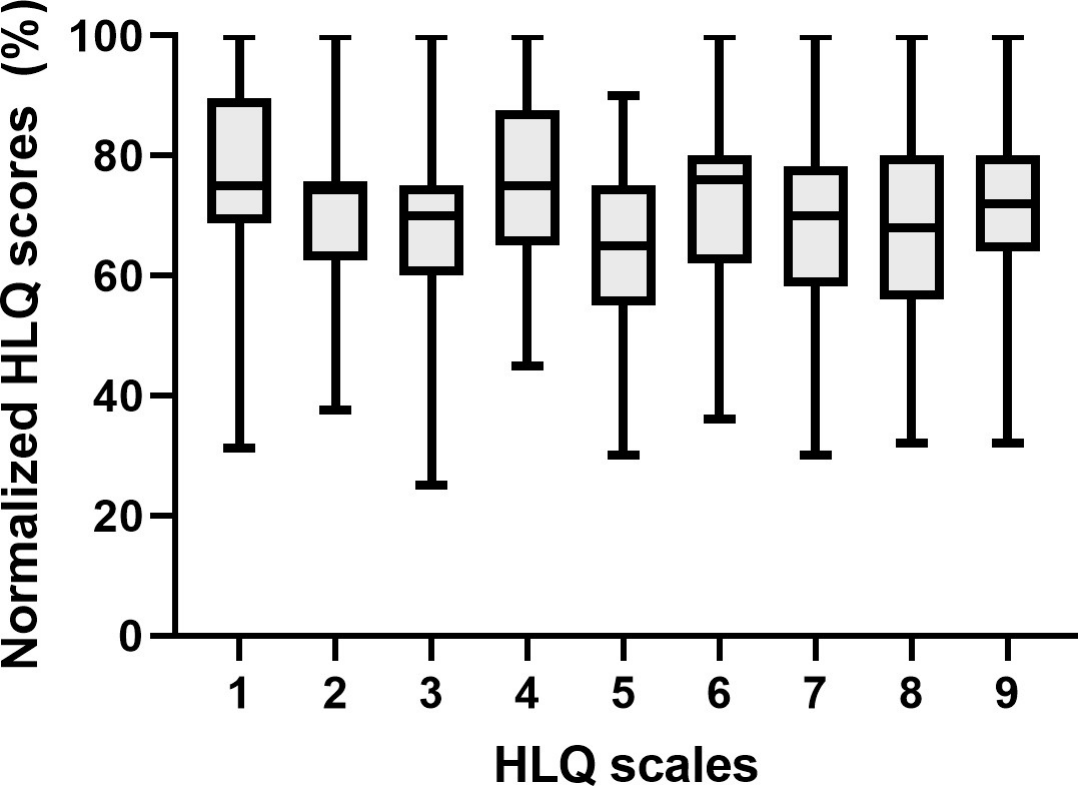
Median of normalized health literacy scores of each scale as assessed by HLQ. Higher scores indicate higher health literacy levels (error bars ≙ ranges). (HLQ: health literacy questionnaire; Scale 1: Feeling understood and supported by healthcare providers (HPS); scale 2: Having sufficient information to manage my health (HIS); scale 3: Actively managing my health (AMH); scale 4: Social support for health (SS); scale 5: Appraisal of health information (CA); scale 6: Ability to actively engage with healthcare providers (AE); scale 7: Navigating the healthcare system (NHS); scale 8: Ability to find good health information (FHI); scale 9: Understand health information well enough to know what to do (UHI)).

In total, 89 in- and outpatients with liver cirrhosis were prospectively studied between January 2019 and November 2020. More than half of the patients were male (57.3%) with a median age of 60 years (IQR 54; 67). Median years of education were 13 (IQR 11; 15). More than 70% of the patients were living in a relationship (71.9%) or had children (77.5%). Most common aetiology of underlying liver disease was chronic alcohol consumption (68.5%). Patients’ disease stages were balanced at study entry (CP A 47%, CP B 37%, CP C 16%) and median MELD score was 14 (9; 19). About two thirds of the patients had a history of ascites (65.2%) and 18.0% a history of OHE. CHE was diagnosed in 27 (32.9%) patients of whom 21 (25.6%) were classified as suffering from MHE. Median HLQ scores ranged between 65–76%. Detailed results of all subscales are displayed in Fig 1. Additional baseline characteristics of the entire cohort are shown in Table 1.

**Table 1:** Baseline characteristics of the entire cohort at study inclusion.

Variables	Total cohort (n = 89)
Age, median (IQR)	60 (54; 67)
Male gender, n (%)	51 (57.3)
Education in years, median (IQR)	13 (11; 15)
Out-patients, n (%)	47 (52.8)
In a relationship, n (%)	64 (71.9)
Children, n (%)	69 (77.5)
Born outside of Germany, n (%)	15 (16.9)
Unemployed/retired/disabled, n (%)	50 (56.2)
Active smoking¸ n (%)	28 (31.5)
History of falls, n (%)	21 (23.6)
**Aetiology of underlying liver disease**	
Chronic alcohol consumption, n (%)	48 (53.8)
Chronic viral hepatitis, n (%)	9 (10.1)
Autoimmune/Cholestatic liver disease, n (%)	3 (3.4)
NAFLD, n (%)	16 (18.0)
Mixed, n (%)	13 (14.6)
**Characteristics of liver cirrhosis**	
Child-Pugh A/B/C, n (%)	42 (47), 33 (37), 14 (16)
MELD score, median (IQR)	14 (9; 19)
Ascites at study inclusion, n (%)	42 (47.2)
History of ascites, n (%)	58 (65.2)
History of OHE, n (%)	16 (18.0)
Presence of varices, n (%)	56 (62.9)
CHE at study entry*, n (%)	27 (32.9)
TIPSS, n (%)	4 (4.5)
**Laboratory values at study inclusion,** median (IQR)	
Sodium, mmol/l	138 (135; 140)
Creatinine, mg/dl	0.87 (0.7; 1.15)
Total bilirubin, g/dl	1.8 (0.82; 4.55)
Albumin	31 (25; 36)
INR	1.3 (1.1; 1.6)
Leukocytes, /nl	6.23 (4.54; 8.27)
Thrombocytes, /nl	108 (88; 171)
**Questionnaires,** median (IQR)	
HLQ:	
• Scale 1	(2.75; 3.5)
• Scale 2	(2.5; 3.0)
• Scale 3	(2.4; 3.0)
• Scale 4	•(2.6; 3.4)
• Scale 5	(2.2; 3.0)
• Scale 6	(3.2; 4.0)
• Scale 7	(3.0; 3.8)
• Scale 8	(2.8; 4.0)
• Scale 9	3.6 (3.2; 4.0)
HDRS	8 (5; 13)

*Tested in 82 patients.

Data are expressed as median and interquartile ranges or as frequencies and percentages.

NAFLD: non-alcoholic fatty liver disease; MELD: model for end-stage liver disease; OHE: overt hepatic encephalopathy; CHE: covert hepatic encephalopathy; TIPPS: transjugular intrahepatic portosystemic stent shunt; HLQ: health literacy questionnaire; HDRS: Hamilton Depression Rating Scale.

### Risk factors of poorer health literacy in patients with liver cirrhosis

First, the correlation of demographic and clinical variables with each score of the sub-scales of the HLQ were assessed by means of univariable analyses. The results of these analyses are displayed in Tables 2 and 3.

**Table 2:** Univariable analyses of risk factors of the HLQ subscales 1–5.

	Scale 1^a^		Scale 2^b^		Scale 3^c^		Scale 4^d^		Scale 5^e^	
	r	p	r	p	r	p	r	p	r	P
Age^#^	0.019	0.863	0.124	0.247	0.116	0.278	0.034	0.751	-0.203	**0.057**
Gender*	0.137	0.200	0.073	0.496	0.204	**0.055**	0.201	**0.059**	0.102	0.340
Education^#^	0.017	0.878	0.096	0.371	0.080	0.455	0.025	0.817	0.114	0.286
In a relationship*	-0.048	0.654	0.220	**0.039**	0.092	0.391	0.195	**0.067**	0.183	**0.087**
Children*	-0.110	0.307	0.110	0.304	0.111	0.301	-0.010	0.924	0.068	0.527
Born outside of Germany*	0.283	**0.007**	0.161	0.133	0.131	0.223	0.094	0.380	0.060	0.576
Unemployed/Retired/disabled*	0.094	0.382	0.058	0.589	0.051	0.635	0.000	0.999	0.045	0.678
Active Smoking*	0.005	0.962	-0.005	0.963	-0.161	0.132	-0.236	**0.026**	-0.061	0.571
History of falls*	-0.123	0.252	-0.290	**0.006**	-0.290	**0.006**	-0,051	0.637	-0.182	**0.088**
Alcoholic cirrhosis*	-0.164	0.125	-0.135	0.208	-0.192	**0.072**	-0.079	0.463	-0.063	0.561
MELD^#^	0.039	0.716	-0.197	**0.064**	-0.286	**0.007**	0.157	0.142	-0.126	0.239
Ascites at study inclusion*	-0.131	0.219	-0.135	0.206	-0.080	0.454	-0.033	0.757	-0.061	0.570
History of ascites*	-0.067	0.533	-0.028	0.793	0.012	0.915	0.036	0.740	-0.224	**0.035**
History of OHE*	0.001	0.991	-0.023	0.827	0.062	0.565	0.077	0.472	-0.087	0.420
Varices*	-0.139	0.195	-0.053	0.621	-0.025	0.818	-0.060	0.589	-0.078	0.470
CHE*	-0.189	**0.089**	-0.203	**0.068**	-0.095	0.395	-0.028	0.803	-0.117	0.295
TIPSS*	0.025	0.818	-0.136	0.204	0.073	0.494	0.118	0.269	0.092	0.389
Sodium^#^	0.049	0.645	0.028	0.792	0.011	0.918	-0.106	0.325	0.189	**0.076**
Albumin^#^	0.113	0.293	0.116	0.279	0.087	0.416	0.042	0.697	0.137	0.202
Thrombocytes^#^	0.044	0.683	0.107	0.318	0.137	0.200	-0.008	0.940	0.114	0.286
HDRS^#^	-0.028	0.792	-0.129	0.230	-0.112	0.300	-0.166	0.122	-0.144	0.181

HDRS: Hamilton Depression Rating Scale; HLQ: Health literacy questionnaire; MELD: Model for end-stage liver disease; CHE: Covert hepatic encephalopathy; TIPPS: Transjugular intrahepatic portosystemic shunt; OHE: Overt hepatic encephalopathy

^#^Spearman’s rank correlation;

*point-biserial correlation

Coding for gender: 0 male, 1 female.

^a^Feeling understood and supported by healthcare providers (HPS)

^b^Having sufficient information to manage my health (HSI)

^c^Actively managing my health (AMH)

^d^Social support for health (SS)

^e^Appraisal of health information (CA)

**Table 3:** Univariable analyses of risk factors of the HLQ subscales 6–9.

Variable	Scale 6^a^		Scale 7^b^		Scale 8^c^		Scale 9^d^	
	r	P	r	p	r	p	r	p
Age^#^	-0.044	0.679	-0.014	0.895	-0.144	0.179	-0.115	0.282
Gender*	0.040	0.712	0.185	**0.083**	0.056	0.599	0.157	0.141
Education^#^	0.131	0.222	0.001	0.994	0.207	**0.051**	0.223	**0.036**
Married*	0.174	0.104	0.157	0.142	0.134	0.212	0.041	0.702
Children*	0.081	0.450	0.066	0.540	0.127	0.236	0.109	0.309
Born outside of Germany*	0.172	0.107	0.133	0.213	0.041	0.705	0.144	0.179
Unemployed/Retired/disabled*	-0.067	0.533	0.103	0.335	-0.039	0.719	-0.092	0.392
Active Smoking*	-0.100	0.351	0.004	0.968	-0.001	0.989	-0.110	0.305
History of falls*	-0.104	0.330	-0.168	0.117	-0.225	**0.034**	-0.255	**0.016**
Alcoholic cirrhosis*	-0.075	0.484	-0.033	0.759	-0.012	0.913	0.100	0.351
MELD^#^	-0.119	0.267	-0.133	0.212	-0.205	**0.054**	-0.217	**0.041**
Ascites at study inclusion*	-0.178	**0.096**	-0.243	**0.022**	-0.155	0.146	-0.194	**0.068**
History of ascites*	-0.180	**0.092**	-0.116	0.280	-0.098	0.363	-0.145	0.175
History of OHE*	-0.029	0.790	0.008	0.942	0.009	0.932	-0.110	0.304
Varices*	-0.175	0.101	-0.038	0.726	-0.016	0.885	-0.105	0.327
CHE*	-0.197	**0.077**	-0.197	**0.076**	-0.202	**0.069**	-0.239	**0.030**
TIPSS*	-0.074	0.489	-0.040	0.711	-0.140	0.191	-0.004	0.970
Sodium^#^	-0.016	0.879	0.118	0.272	0.081	0.448	0.001	0.995
Albumin^#^	0.133	0.212	0.176	0.099	0.104	0.333	0.149	0.163
Thrombocytes^#^	0.081	0.450	0.088	0.410	0.077	0.471	0.159	0.136
HDRS^#^	-0.328	**0.002**	-0.271	**0.011**	-0.194	**0.070**	-0.121	0.260

HDRS: Hamilton Depression Rating Scale; HLQ: Health literacy questionnaire; MELD: Model for end-stage liver disease; CHE: Covert hepatic encephalopathy; TIPPS: Transjugular intrahepatic portosystemic shunt; OHE: Overt hepatic encephalopathy

^#^Spearman’s rank correlation;

*point-biserial correlation

Coding for gender: 0 male, 1 female.

^a^Ability to actively engage with healthcare providers (AE) (5 items),

^b^Navigating the healthcare system (NHS) (6 items),

^c^Ability to find good health information (FHI) (5 items),

^d^Understanding health information well enough to know what to do (UHI) (5 items).

Variables with p < 0.1 were subsequently considered in a multivariable linear regression model for each sub-scale. The results of these analyses are presented in Tables 4 and 5.

**Table 4:** Multivariable analyses of risk factors of the HLQ subscales 1–5, which were found significant in univariable analyses.

	Scale 1^a^		Scale 2^b^		Scale 3^c^		Scale 4^d^		Scale 5^e^	
	β	p	β	p	β	p	β	p	β	P
Gender					0.259	0.009				
Active smoking							-0.236	0.026		
History of falls			-0.270	0.012	-0.277	0.006				
MELD			-0.220	0.040	-0.284	0.004				
History of ascites									-0.224	0.035
CHE	-0.189	0.089								

HLQ: Health literacy questionnaire; MELD: Model for end-stage liver disease; CHE: Covert hepatic encephalopathy.

Coding for gender: 0 male, 1 female.

Multivariable linear regression model with a stepwise variable selection included (only the significant variables are displayed):

^a^CHE, born outside of Germany; R^2^ statistic for the model = 0.036

^b^MELD, CHE, history of falls, married; R^2^ statistic for the model = 0.135

^c^MELD, history of falls, alcoholic aetiology of liver cirrhosis, gender; R^2^ statistic for the model = 0.219

^d^Active smoking, relationship status, gender; R^2^ statistic for the model = 0.056

^e^Age, sodium, history of falls, history of ascites, relationship status; R^2^ statistic for the model = 0.050

**Table 5:** Multivariable analyses of risk factors of HLQ subscales 6–9, which were found significant in univariable analyses.

Variable	Scale 6^a^		Scale 7^b^		Scale 8^c^		Scale 9^d^	
	β	P	β	p	β	p	β	p
Years of education							0.221	0.041
History of falls					-0.218	0.045	-0.251	0.021
MELD					-0.228	0.036		
HDRS	-0.275	0.013	-0.245	0.027				

HLQ: Health literacy questionnaire; MELD: Model for end-stage liver disease; HDRS: Hamilton Depression Rating Scale.

Multivariable linear regression model with a stepwise variable selection included (only the significant variables are displayed):

^a^HDRS, CHE, history of ascites; R^2^ statistic for the model = 0.076

^b^HDRS, albumin serum levels, CHE, ascites at study inclusion, gender; R^2^ statistic for the model = 0.060

^c^Years of education, HDRS, MELD, CHE, history of falls; R^2^ statistic for the model = 0.108

^d^Years of education, MELD, CHE, history of falls, ascites at study inclusion; R^2^ statistic for the model = 0.134

In multivariable analyses, the presence of CHE (standardized β coefficient = -0.189, p = 0.089) seemed to be associated with a poorer feeling to be understood and supported by healthcare providers (scale 1). History of falls (standardized β coefficient = -0.270, p = 0.012) and higher MELD score (standardized β coefficient = -0.220, p = 0.040) were independent factors associated with a feeling of not having sufficient information to manage the respective persons health (scale 2). Again, a history of falls (standardized β coefficient = -0.277, p = 0.006) and higher MELD score (standardized β coefficient = -0.284, p = 0.004) as well as male gender (standardized β coefficient = 0.259, p = 0.009) were associated with poorer results in the sub-scale of actively managing my health (scale 3). Active smoking (standardized β coefficient = -0.236, p = 0.026) was the only factor associated with a feeling of poorer social support for health (scale 4). A history of ascites (standardized β coefficient = -0.224, p = 0.035) was the only independent risk factors of poorer appraisal of health information (scale 5). Depressive symptoms, expressed as higher scores in HDRS, were independently associated with a poorer ability to actively engage with healthcare providers (scale 6, standardized β coefficient = -0.275, p = 0.013) and a poorer ability to navigate the healthcare system (scale 7, standardized β coefficient = -0.245, p = 0.027). A higher MELD score (standardized β coefficient = -0.228, p = 0.036) and a history of falls (standardized β coefficient = -0.218, p = 0.045) were associated with a poorer ability to find good health information (scale 8). Last, a shorter education period (standardized β coefficient = 0.221, p = 0.041) and again a history of falls (standardized β coefficient = -0.251, p = 0.021) were risk factors for poorer results in scale 9 (understand health information well enough to know what to do), respectively.

## Discussion

In the present study, we were able to demonstrate that several factors seem to have an impact on health literacy in patients with liver cirrhosis. Notably, we identified some potentially modifiable and preventable factors such as CHE, ascites, falls or depressive symptoms. These findings may provide healthcare professionals with detailed information on which patients may be at increased risk for poorer health literacy.

Health literacy is an often overlooked factor in chronic disease management and needs integration in best practices to improve health outcomes [23, 24]. Patients with liver cirrhosis suffer from an end-stage chronic disease and are therefore at risk for poor health literacy. However, despite the acknowledged importance of health literacy in patients with liver cirrhosis there is still scarce data available. One potential explanation may be that until recently no standardized tools to measure and quantify health literacy were available. Therefore, the HLQ was developed as a validated tool for the assessment of health literacy in all kinds of patients [5].

In the present study, we enrolled 89 in- and outpatients with liver cirrhosis in a tertiary care hospital in Germany and assessed their heath literacy using the validated German version of the HLQ [25]. HLQ does not provide one overall summative score and no cut offs for low or high levels of health literacy have been defined in detail [5]. Each scale needs to be interpreted by itself and scores need to be referenced to previous data. In the present study, the lowest score was found for scale 5 (CA, 65% approval), while the highest score was found for scale 6 (AE, 76% approval). The only comparable study in this setting was conducted by Grydgaard et al. in a Danish tertiary care centre, but HLQ was only assessed for scales 4 (SS), 6 (AE) and 9 (UHI) [16]. They found a normalized mean approval of 77.5%, 78% and 76% for scale 4 (SS), 6 (AE) and 9 (UHI), respectively, and assessed rates ≤75% as an indicator of low levels of health literacy, providing a benchmark for future studies. Considering this cut off, the overall level of health literacy in our cohort was low with a median approval of all scales of 72% (range 65% - 76%). Only one scale—ability to actively engage with healthcare providers (scale 6)—reached a median approval of 76% indicating sufficient health literacy. Overall, the HLQ scores in our study are slightly lower than those of the Danish patients. A potential explanation for this finding may be the composition of the cohorts. In the Danish cohort only outpatients with liver cirrhosis were recruited, while we investigated a mixed cohort consisting of compensated as well as decompensated hospitalized patients. The hypothesis that especially patients with chronic diseases like liver cirrhosis are at high risk for poor health literacy is supported by studies investigating other chronic diseases. A meta-analysis conducted by Taylor et al. evaluated 29 studies with a total number of 18,300 patients, assessing health literacy in adults with chronic kidney disease. They concluded that 25% of patients with chronic kidney disease have limited health literacy, which could impact clinical outcomes [26].

The identification of patients with poor health literacy may be challenging in routine clinical practice. To get a better insight into the determinants of poor health literacy and to inform healthcare providers on potential high-risk groups, we identified several risk factors for poorer scores in the different subgroups of the HLQ by performing linear regression models. We found that poor liver function, as defined by higher MELD scores, independently correlated with a poorer ability to find good health information, manage one’s own health and a feeling of not having sufficient information to manage health (scales 2 (HIS), 3 (AMH) and 8 (FHI)). This is a worrisome finding indicating that especially patients with an advanced stage of their liver cirrhosis are restricted in their health literacy. In accordance, we found a history of ascites to be associated with difficulties with appraisal of health information. Taken together, these findings should encourage healthcare providers to better support patients with advanced liver cirrhosis in their health literacy and to focus on this high-risk group.

We could identify several modifiable and preventable factors that may impact on health literacy. Most striking was a history of falls as it correlated with having insufficient information on actively managing one’s own health, ability to find good health information and difficulties understanding health information (scale 2, 3, 8 and 9). Falls are common in patients with liver cirrhosis and could be figuratively considered as an indicator of higher morbidity and frailty in these patients [27–29]. In addition, there is ample evidence that the occurrence of falls is a major determinant of poorer health related quality of life in patients with liver cirrhosis [30, 31]. The occurrence of falls is complex and triggered by a combination of reduced physical reserve and cognitive decline, potentially caused by CHE, which all contribute to frailty [32]. Unfortunately, we did not screen our patients for frailty and therefore it is only a hypothesis that the occurrence of falls is a possible surrogate for frailty. Finally, preventing morbidity and frailty in patients with liver cirrhosis thereby reducing the occurrence of falls might have a positive impact on health literacy.

Another modifiable or preventable factor to improve health literacy was the presence of CHE. CHE correlated with the feeling of patients being poorly understood and supported by healthcare providers (scale 1). This finding is not surprising since early signs of emerging HE are slight changes of personality, probably resulting in unfounded mistrust towards healthcare providers. CHE is a reversible cognitive dysfunction, which can be easily treated with non-absorbable disaccharides (e.g. lactulose) and may therefore be an accessible target to improve this part of health literacy [33]. However, it has to be mentioned that we cannot prove causality and this hypothesis has to be proven in future prospective interventional trials. Nevertheless, our findings should increase the awareness of healthcare providers regarding the negative effects of CHE. Recent evidence suggests that testing for CHE is often neglected in routine clinical practice, although this is recommended in current guidelines [19, 34].

Depressive symptoms are common in patients with liver cirrhosis and adversely affect clinical outcomes [35–37]. We were able to demonstrate that depressive symptoms according to the HDRS had detrimental effects on healthy literacy by compromising the ability to engage with healthcare providers and to navigate in the healthcare system (scale 6 and 7). This finding underlines the importance of searching for signs of depression in routine clinical practice to offer interdisciplinary treatment programs to affected patients.

Last, we found gender-based differences on how women and men deal with their disease. Men seemed to be less confident in managing their health than women (scale 3). This is a surprising finding, since especially studies investigating health related quality of life in patients with chronic liver disease or cirrhosis indicated that quality of life is remarkably impaired in women [15, 38]. Our current finding may be explained by the hypothesis that women may have a greater interest in personal health.

Our study has some limitations that need to be acknowledged. First, the concept of our study was a cross-sectional design. Therefore, we were only able to identify potential associations between different variables and poorer health literacy and causality has to be proven in future studies. Moreover, due to our cross-sectional study design we are unable to assess whether the occurrence of complications of cirrhosis leads to poorer health literacy or whether poorer health literacy leads to a poorer prognosis and more rapid disease progression. Therefore, our results have to be strictly interpreted in the context of the study design. Additionally, we are unable to assess whether measures like prevention of falls or treatment of depressive symptoms or CHE would result in an improved health literacy. We did not study patients with any malignancy. Therefore, our findings may not be generalizable to all patients with liver cirrhosis. Another limitation that has to be acknowledged is the comparably small sample size of this study. Additionally, our complete data analyses were exploratory, and we did not adjust for multiple testing. Therefore, our findings should be interpreted with caution and need validation in future, larger multicentre studies to ensure precision in estimates. Last, we only assessed depressive symptoms using the HDRS and did not search for other mental disorders like e.g. anxiety disorder. Consequently, we cannot exclude a possible influence of other mental disorders apart from depression on health literacy.

In conclusion, we were able to demonstrate that several factors seem to have an impact on health literacy in patients with liver cirrhosis. These findings provide healthcare professionals with detailed information on patients at high risk for poor health literacy. Addressing modifiable and preventable factors may improve health literacy.

## Abbreviations

ACLF: Acute-on-chronic liver failure
AE: Ability to actively engage with healthcare providers
AL: Acute liver failure
AMH: Actively managing my health
CA: Appraisal of health information
CCM: Cirrhosis Centre Mainz
CHE: covert hepatic encephalopathy
CP: Child-Pugh
FHI: Ability to find good health information
Gold: Global Initiative for Chronic Obstructive Lung Disease
HDRS: Hamilton Depression Rating Scale
HE: hepatic encephalopathy
HE1: hepatic encephalopathy grade 1
HLQ: Health Literacy Questionnaire
HPS: Feeling understood and supported by healthcare providers
HSI: Having sufficient information to manage my health
IQR: interquartile ranges
LTT: line tracing test
MELD: Model of end-stage liver disease
MHE: minimal hepatic encephalopathy
NAFLD: non-alcoholic fatty liver disease
NCT-A: Number connection test-A
NCT-B: number connection test-B
NHS: Navigating the healthcare system
NYHA: New York Heart Association
OHE: overt hepatic encephalopathy
PHES: Portosystemic Hepatic Encephalopathy Score
PSE: Portosystemic encephalopathy
SDT: serial dotting test
SS: Social support for health
TIPSS: Transjugular intrahepatic portosystemic stent shunt
UHI: Understanding health information well enough to know what to do
